# The microbiota–gut–brain axis regulates motivation for exercise

**DOI:** 10.1002/mco2.304

**Published:** 2023-06-13

**Authors:** Wenhuan Sun, Zhenjiang Bai, Fangfang Zhou

**Affiliations:** ^1^ Pediatric Intensive Care Unit Children's Hospital of Soochow University Suzhou China; ^2^ Institutes of Biology and Medical Science Soochow University Suzhou China

1

In a recent study published in *Nature*, Dohnalová et al.^1^ found that the motivation for long‐term physical activity in mice is not entirely driven by the brain, it is also regulated by gut microbes. They reported that mice with proficient running capabilities greatly benefitted from two types of gut bacteria. Furthermore, it was discovered that these bacteria stimulate enteric nerves by generating fatty acid amides (FAA), which are diminutive molecule metabolites that elevate the ventral striatum of the brain, subsequently heightening the levels of dopamine in the body, thus promoting a propensity to engage in physical exercise. This discovery sheds light on the gut‐to‐brain pathway, explaining why some bacteria boost athletic performance (Figure 1).

**FIGURE 1:**
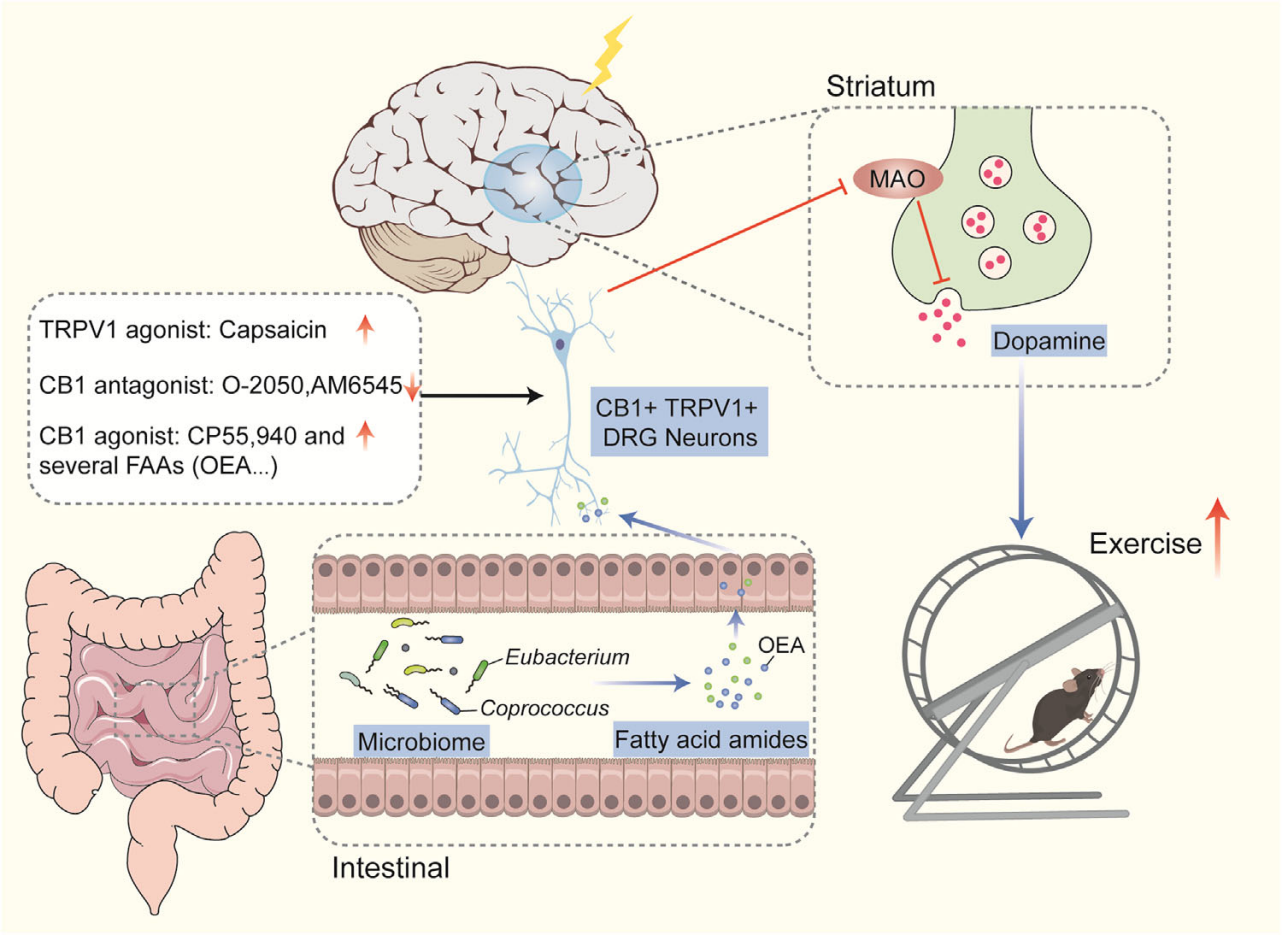
Gut microbiota containing *Eubacterium* and *Coprococcus* can secrete fatty acid amides, like *N*‐oleoylethanolamide (OEA), which stimulate CB1 and TRPV1 receptors in the dorsal root ganglia. This prompts a decrease in monoamine oxidase (MAO) expression in the brain's striatum region, leading to more dopamine release and promoting exercise desire. The CB1 and TRPV1 receptors can be targeted to facilitate or block gut–brain–motor signaling axis. Brain cartoon images used were obtained from Scidraw.io.

Like other mammals, humans are inhabited by trillions of microbes, including bacteria, viruses, and fungi, collectively known as commensal flora. In a sense, “human” is a multicomponent complex composed of the human body and symbiotic flora. There are large numbers of parasitic microorganisms in the human gut. Gut microorganisms affect human obesity, enteritis, autoimmune diseases, responses to cancer treatment drugs, and even life expectancy. The gut microbiota influences the physiological characteristics of the host during the earliest stages of life. Alterations in gut microbiota may damage key processes in the development of organ systems, including the brain. Accumulating evidence has revealed the degree of interdependence between humans and human gut microbiota, emphasizing the importance of the brain–gut axis.^2^

Regular exercise is increasingly being prescribed as an adjunctive therapy for a variety of human diseases.^3^ Poor fitness and physical inactivity are leading causes of chronic non‐communicable diseases (CNCDs) such as heart disease, stroke, type 2 diabetes, chronic respiratory disease, and some cancers,^4^, ^5^ highlighting the urgent need for targeted efforts to reverse this trend. To identify factors that determine exercise performance, the authors^1^ focused on genome sequences, gut bacterial species, blood metabolites, and other data from multiple mouse models with different genetic backgrounds and assessed their relative contribution to exercise performance. They then measured the daily voluntary wheel movement and endurance of the mice. These experimental data were then analyzed by machine learning to find the correlation between the physiological parameters and the exercise ability of mice in order to explain the huge differences in running performance between individuals. The team was surprised to find that genes accounted for only a small portion of these differences in athletic performance, whereas the composition of the bacterial community alone predicted running performance with high accuracy, suggesting that gut bacteria drive athletic performance. Moreover, depletion of gut bacteria through antibiotic treatment reduces exercise tolerance. Transferring the gut microbiota from high‐performing mice to germ‐free mice increased the functioning capacity of the recipients to a level that matched that of the microbiome sample donors. Furthermore, the authors have disclosed that the gut microbiota exerts an impact on exercise performance by means of striatal activation, as evidenced by the results of single‐nucleus RNA‐sequencing (RNA‐seq) analysis of the striatum.

Using different classes of antibiotics, the authors identified two gut bacteria that were strongly associated with better exercise performance: *Eubacterium* and *Coprococcus*. Furthermore, utilizing untargeted metabolomics, the metabolites in the gut linked to these particular intestinal bacteria were identified as FAAs, notably *N*‐oleoylethanolamide (OEA). FAA bind to the endogenous cannabinoid receptor CB1 and stimulate the dorsal root ganglia (DRG) to express the vanilloid receptor TRPV1 in the gut, which connects to the brain through the spine. During exercise, the intestinal sensory nerves expressed CB1 receptors are stimulated, leading to the inhibition of monoamine oxidase (MAO) expression, which degrades dopamine and other neurotransmitter molecules in the ventral striatum region of the brain, prompting the release of neurotransmitter dopamine. The striatum is a key node in the brain's reward and motivation network; therefore, higher dopamine levels in this region during exercise can increase the desire to exercise and improve athletic performance. Furthermore, the authors discovered that bouts of dopamine triggered by physical exercise activate particular neuronal subpopulations in the striatum area of the brain.

Inhibition of dopamine signaling reduces exercise tolerance, suggesting that exercise‐induced dopamine surges and subsequent striatal neuronal activity are critical for maintaining a willingness to exercise. The activation of dopamine signaling restored the exercise performance of antibiotic‐treated mice, as the authors have noted. Furthermore, the authors have indicated that the expression of the dopamine‐degrading enzyme, MAO, decreased in the exercising mice, but not in those where the microbiota was depleted, indicating that the latter's sluggish dopamine response was due to MAO‐driven dopamine turnover. Restoration of the gut microbiome, inhibition of MAO, and increased artificial dopamine signaling in the striatum are adequate measures for reinstating exercise capacity in mice that lack gut bacteria. Additionally, the microbiota's influence on dopamine signaling requires the involvement of intestinal neurosensory neurons that expressed TRPV1 and CB1. Most importantly, the authors have demonstrated that microbiota‐derived FAAs promote exercise‐induced neuronal activation, striatal dopamine release, and enhance motor performance.

In conclusion, the work by Dohnalová et al.^1^ has important implications for our understanding of exercise physiology and the relationship between the gut microbiota and exercise behavior. By uncovering a potential mechanism by which the gut microbiota enhances the body's desire to exercise, this study opens up new avenues for research into the gut–brain axis and exercise. Furthermore, the finding that the gut microbiota also influences the “Runner's High” phenomenon through the release of endocannabinoids, which were known to have analgesic properties, provide insights into how the gut microbiota and peripheral CB1 signaling can regulate pain sensitivity during exercise. This gut‐to‐brain pathway may have evolved to link nutrient availability and the state of the gut bacterial population with readiness for prolonged exercise. Nevertheless, certain crucial matters are yet to be resolved, such as the signaling mechanism originating from sensory neurons that govern the expression of MAO in the striatum. The discoveries made by the authors allude to fresh insights concerning the factors that may augment physical prowess. Should these revelations be substantiated in humans, they prompt the inquiry of whether manipulating gut microbiota or adhering to certain dietary regimens could ameliorate cognitive functions linked to exercise in a personalized manner.

## AUTHOR CONTRIBUTIONS

Wenhuan Sun drew the images and wrote the article. Fangfang Zhou and Zhenjiang Bai provided modifications. All authors have read and approved the final manuscript.

## CONFLICT OF INTEREST STATEMENT

The authors declare they have no conflicts of interest.

## ETHICS STATEMENT

No ethical approval was necessary for this work.

## Data Availability

No data was used for the research described in this highlight.
